# Modelling pathological spread through the structural connectome in the frontotemporal dementia clinical spectrum

**DOI:** 10.1093/brain/awae391

**Published:** 2024-11-29

**Authors:** Federica Agosta, Silvia Basaia, Edoardo G Spinelli, Federica Facente, Laura Lumaca, Alma Ghirelli, Elisa Canu, Veronica Castelnovo, Elisa Sibilla, Chiara Tripodi, Fabiola Freri, Giordano Cecchetti, Giuseppe Magnani, Francesca Caso, Federico Verde, Nicola Ticozzi, Vincenzo Silani, Paola Caroppo, Sara Prioni, Cristina Villa, Lucio Tremolizzo, Ildebrando Appollonio, Ashish Raj, Massimo Filippi

**Affiliations:** Neuroimaging Research Unit, Division of Neuroscience, IRCCS San Raffaele Scientific Institute, 20132 Milan, Italy; Vita-Salute San Raffaele University, 20132 Milan, Italy; Neurology Unit, IRCCS San Raffaele Scientific Institute, 20132 Milan, Italy; Neuroimaging Research Unit, Division of Neuroscience, IRCCS San Raffaele Scientific Institute, 20132 Milan, Italy; Neuroimaging Research Unit, Division of Neuroscience, IRCCS San Raffaele Scientific Institute, 20132 Milan, Italy; Vita-Salute San Raffaele University, 20132 Milan, Italy; Neurology Unit, IRCCS San Raffaele Scientific Institute, 20132 Milan, Italy; Epione Research Team, Inria Center of Université Côte d’Azur, 06560 Biot-Sophia Antipolis, France; Neuroimaging Research Unit, Division of Neuroscience, IRCCS San Raffaele Scientific Institute, 20132 Milan, Italy; Neuroimaging Research Unit, Division of Neuroscience, IRCCS San Raffaele Scientific Institute, 20132 Milan, Italy; Vita-Salute San Raffaele University, 20132 Milan, Italy; Neurology Unit, IRCCS San Raffaele Scientific Institute, 20132 Milan, Italy; Neuroimaging Research Unit, Division of Neuroscience, IRCCS San Raffaele Scientific Institute, 20132 Milan, Italy; Neuroimaging Research Unit, Division of Neuroscience, IRCCS San Raffaele Scientific Institute, 20132 Milan, Italy; Neuroimaging Research Unit, Division of Neuroscience, IRCCS San Raffaele Scientific Institute, 20132 Milan, Italy; Neuroimaging Research Unit, Division of Neuroscience, IRCCS San Raffaele Scientific Institute, 20132 Milan, Italy; Neuroimaging Research Unit, Division of Neuroscience, IRCCS San Raffaele Scientific Institute, 20132 Milan, Italy; Neuroimaging Research Unit, Division of Neuroscience, IRCCS San Raffaele Scientific Institute, 20132 Milan, Italy; Vita-Salute San Raffaele University, 20132 Milan, Italy; Neurology Unit, IRCCS San Raffaele Scientific Institute, 20132 Milan, Italy; Neurophysiology Service, IRCCS San Raffaele Scientific Institute, 20132 Milan, Italy; Neurology Unit, IRCCS San Raffaele Scientific Institute, 20132 Milan, Italy; Neurology Unit, IRCCS San Raffaele Scientific Institute, 20132 Milan, Italy; Department of Neurology and Laboratory of Neuroscience, IRCCS Istituto Auxologico Italiano, 20122 Milan, Italy; Department of Neurology and Laboratory of Neuroscience, IRCCS Istituto Auxologico Italiano, 20122 Milan, Italy; ‘Dino Ferrari’ Center, Department of Pathophysiology and Transplantation, Università degli Studi di Milano, 20122 Milan, Italy; Department of Neurology and Laboratory of Neuroscience, IRCCS Istituto Auxologico Italiano, 20122 Milan, Italy; ‘Dino Ferrari’ Center, Department of Pathophysiology and Transplantation, Università degli Studi di Milano, 20122 Milan, Italy; Unit of Neurology Neuropathology, Fondazione IRCCS Istituto Neurologico Carlo Besta, 20133 Milan, Italy; Unit of Neurology Neuropathology, Fondazione IRCCS Istituto Neurologico Carlo Besta, 20133 Milan, Italy; Unit of Neurology Neuropathology, Fondazione IRCCS Istituto Neurologico Carlo Besta, 20133 Milan, Italy; Neurology Unit, ‘San Gerardo’ Hospital and University of Milano-Bicocca, 20900 Monza, Italy; Neurology Unit, ‘San Gerardo’ Hospital and University of Milano-Bicocca, 20900 Monza, Italy; Department of Radiology, University of California San Francisco, San Francisco, CA 94107, USA; Neuroimaging Research Unit, Division of Neuroscience, IRCCS San Raffaele Scientific Institute, 20132 Milan, Italy; Vita-Salute San Raffaele University, 20132 Milan, Italy; Neurology Unit, IRCCS San Raffaele Scientific Institute, 20132 Milan, Italy; Neurophysiology Service, IRCCS San Raffaele Scientific Institute, 20132 Milan, Italy

**Keywords:** connectomics, frontotemporal dementia, network spreading

## Abstract

The ability to predict the spreading of pathology in patients with frontotemporal dementia (FTD) is crucial for early diagnosis and targeted interventions. In this study, we examined the relationship between network vulnerability and longitudinal progression of atrophy in FTD patients, using the network diffusion model (NDM) of the spread of pathology.

Thirty behavioural variant FTD (bvFTD), 13 semantic variant primary progressive aphasia (svPPA), 14 non-fluent variant primary progressive aphasia (nfvPPA) and 12 semantic behavioural variant FTD (sbvFTD) patients underwent longitudinal T_1_-weighted MRI. Fifty young controls (20–31 years of age) underwent multi-shell diffusion MRI scan. An NDM was developed to model progression of FTD pathology as a spreading process from a seed through the healthy structural connectome, using connectivity measures from fractional anisotropy and intracellular volume fraction in young controls. Four disease epicentres were initially identified from the peaks of atrophy of each FTD variant: left insula (bvFTD), left temporal pole (svPPA), right temporal pole (sbvFTD) and left supplementary motor area (nfvPPA). Pearson’s correlations were calculated between NDM-predicted atrophy in young controls and the observed longitudinal atrophy in FTD patients over a follow-up period of 24 months. The NDM was then run for all 220 brain seeds to verify whether the four epicentres were among those that yielded the highest correlation.

Using the NDM, predictive maps in young controls showed progression of pathology from the peaks of atrophy in svPPA, nfvPPA and sbvFTD over 24 months. svPPA exhibited early involvement of the left temporal and occipital lobes, progressing to extensive left hemisphere impairment. nfvPPA and sbvFTD spread in a similar manner bilaterally to frontal, sensorimotor and temporal regions, with sbvFTD additionally affecting the right hemisphere. Moreover, the NDM-predicted atrophy of each region was positively correlated with longitudinal real atrophy, with a greater effect in svPPA and sbvFTD. In bvFTD, the model starting from the left insula (the peak of atrophy) demonstrated a highly left-lateralized pattern, with pathology spreading to frontal, sensorimotor, temporal and basal ganglia regions, with minimal extension to the contralateral hemisphere by 24 months. However, unlike the atrophy peaks observed in the other three phenotypes, the left insula did not show the strongest correlation between the estimated and real atrophy. Instead, the bilateral superior frontal gyrus emerged as optimal seeds for modelling atrophy spread, showing the highest correlation ranking in both hemispheres.

Overall, NDM applied on the intracellular volume fraction connectome yielded higher correlations relative to NDM applied on fractional anisotropy maps. The NDM implementation using the cross-sectional structural connectome is a valuable tool to predict patterns of atrophy and spreading of pathology in FTD clinical variants.

## Teaser

Agosta *et al.* examine the relationship between network vulnerability and atrophy progression in FTD, using the Network Diffusion Model of pathology spread. They show that the model can accurately predict atrophy evolution, showing significant correspondence with real data, particularly for focal variants such as svPPA and sbvFTD.

## Introduction

The most common neurodegenerative conditions are characterized by a pathological deposition of misfolded proteins throughout the CNS. This process is believed to proceed mostly in stereotyped patterns, as described by histopathological staging systems of Alzheimer’s disease (AD),^1^ Parkinson’s disease (PD),^2^ frontotemporal dementia (FTD)^3^ and amyotrophic lateral sclerosis (ALS).^4^ However, post-mortem findings obtained from patients affected by these diseases cannot provide any information regarding the dynamic evolution of molecular alterations and their spreading through different brain regions. A mechanism of trans-neuronal transmission of aggregate-prone proteins in a prion-like fashion is an intriguing hypothesis supported by *in vivo* and *in vitro* findings,^5,6^ in which network connectivity might influence the usual pathway of pathology spread.^7^

The network diffusion model (NDM) has been used to model mathematically the progression of pathology spreading across the human brain connectome in this context. The NDM was introduced by Raj *et al*.,^8^ who generalized the ‘network heat equation’^9^ to describe the progression of any pathology from a high concentration to a lower concentration, until reaching an equilibrium state. The principal aim of the NDM is to explore selective vulnerability and disease progression through the healthy connectome, using a quantitative network-based model of pathology spread originating from a single regional seed. In fact, NDM assumes that the transmission of misfolded proteins spreading along neuronal pathways can be modelled using a diffusive mechanism mediated by the brain connectivity network.^8,10^ Given that computation of the NDM differential equation involves an eigenvector decomposition, previous studies found that each eigenmode represents spatial patterns that have a strong resemblance to known patterns of brain damage in different dementias, including AD, the behavioural variant of FTD (bvFTD),^8^ ALS^11^ and Huntington’s disease.^10^ Of note, the NDM also showed significant predictability on longitudinal progression of atrophy and evolution of hypometabolism in AD.^12^ In a subsequent study, the NDM was modified by introducing a directional connectome, in which the direction of the connections is considered.^13^ This method was applied to progressive supranuclear palsy and was shown to explain the topographical distribution of brain damage in progressive supranuclear palsy more accurately than non-directional transmission, strongly supporting a trans-neuronal transmission model of tau pathology in this clinical presentation.^13^ Finally, the NDM was also used ‘backwards’, i.e. to estimate the seed region where the pathology starts to spread in patients with AD^14^ and in PD,^15^ therefore representing the most likely disease epicentre.

Given these premises, implementation of the NDM to cross-sectional structural connectome data is a valuable tool to predict future patterns of atrophy and spreading of pathology in neurodegenerative disorders, simulating the hypothetical spread of disease-causing proteinopathy into the network. However, the validation of this model necessarily involves a correlation between empirical MRI longitudinal data and data predicted by the NDM, similar to previous studies assessing connectivity-based prediction models.^16,17^

In contrast to the pathologically homogeneous AD, clinical variants of FTD are known to harbour highly variable neuropathological underpinnings,^18^ providing an ideal framework in which to evaluate NDM across different proteinopathies. The aim of this study was to test and directly compare the performance of NDM across the clinical spectrum of FTD presentations that are known to be related to TAU or TDP-43 pathologies, including bvFTD and the semantic (svPPA) and non-fluent variants (nfvPPA) of primary progressive aphasia. Moreover, we also focused on the recently systematized syndrome of the semantic behavioural variant of FTD (sbvFTD),^19^ in order to provide further characterization of this poorly described clinical presentation. We hypothesized that fitting of the NDM to longitudinal atrophy patterns will show variability in NDM performance across different clinical presentations, reflecting the heterogeneity in disease progression and underlying neuropathology among FTD subtypes.

## Materials and methods

### Participants

A total of 283 patients with a suspected diagnosis of FTD disorders were referred between June 2017 and January 2023 to the Neurology Unit of IRCCS San Raffaele Hospital in Milan to perform an optimized diagnostic protocol^20^ including neurological work-up, neuropsychological evaluation and 3 T brain MRI, as part of their diagnostic work-up. Following this multidisciplinary evaluation, 236 patients were confirmed as affected by an FTD-related syndrome, with the remaining patients being excluded by evidence of Alzheimer’s pathology at lumbar puncture or absence of signs of neurodegeneration at MRI/18-fluorodeoxyglucose PET. According to current clinical criteria, patients were diagnosed with bvFTD (*n* = 63),^21^ svPPA (*n* = 25),^22^ nfvPPA (*n* = 21),^22^ sbvFTD (*n* = 15),^19^ motor neuron disease (*n* = 67)^23^ or atypical parkinsonism (progressive supranuclear palsy or corticobasal syndrome) (*n* = 45).^24,25^ Patients who received a clinical diagnosis of bvFTD, sbvFTD, nfvPPA or svPPA were evaluated for inclusion in the present longitudinal study. To mitigate sources of sample heterogeneity, after screening for known pathogenic mutations (see below for details), six patients with a pathological expansion in the *C9orf72* gene and 14 with known pathogenic variants on other FTD-related genes (i.e. 12 *GRN* and 2 *MAPT*) were identified and excluded. Six FTD patients (i.e. one bvFTD, four nfvPPA and one svPPA) who demonstrated a high cerebrovascular burden or motion artefacts on MRI were also excluded. Longitudinal follow-up visits were planned at 6, 12, 18 and 24 months (Supplementary Table 1), and patients who were able to perform at least one follow-up scheduled visit were included in the present study. The final cohort included 69 patients with sporadic FTD, including 30 bvFTD, 14 nfvPPA, 13 svPPA and 12 sbvFTD (Table 1). A subsample of 39 patients (20 bvFTD, 7 svPPA, 7 nfvPPA and 5 sbvFTD) also underwent lumbar puncture for quantification of CSF biomarkers (Aβ_42_, tTau and pTau); no patients showed an AD-like biomarker profile based on the pTau/Aβ_42_ ratio. Diagnoses were verified at each time point considered in the study. For bvFTD and sbvFTD patients, the diagnosis was confirmed at the last follow-up (after 24 months). In the svPPA group, 9 of 13 patients remained stable, whereas 4 transitioned to a semantic dementia diagnosis. Furthermore, at the last follow-up, 9 of 14 nfvPPA patients remained stable, whereas 5 transitioned to dementia. In addition, 50 young healthy controls (age range 20–30 years, 23 females) were recruited, to represent a ‘reference’ healthy connectome for the construction of the NDM (Supplementary Tables 2 and 3).

**Table 1:** Demographic and main clinical characteristics of included subjects

Characteristic	bvFTD	svPPA	nfvPPA	sbvFTD
	*n* = 30	*n* = 13	*n* = 14	*n* = 12
Age at MRI, years	67.14 ± 7.63 (46.51–79.76)	66.35 ± 7.99 (49.91–75.31)	71.38 ± 9.75 (51.57–83.87)	63.16 ± 8.99 (48.36–77.15)
Sex, male/female	17/13	6/7	5/9	9/3
Education, years	10.23 ± 3.48 (3.00–18.00)	13.08 ± 3.57 (5.00–17.00)	10.54 ± 6.01 (5.00–22.00)	9.42 ± 3.06 (5.00–13.00)
Disease duration, years	3.51 ± 2.05 (0.56–8.97)	4.66 ± 2.20 (1.00–9.96)	3.15 ± 1.61 (1.17–5.67)	4.16 ± 2.94 (1.46–10.42)
CDR	0.96 ± 0.82 (0.00–3.00)	0.67 ± 0.56 (0.00–2.00)	0.58 ± 0.73 (0.00–2.00)	0.59 ± 0.30 (0.00–1.00)
CDR plus NACC FTLD	7.50 ± 5.39 (1.00–23.00)	3.94 ± 4.30 (1.00–14.00)	5.00 ± 4.40 (0.50–14.50)	5.23 ± 2.54 (2.50–11.00)
CDR-sb	5.44 ± 4.25 (1.00–17.00)	3.06 ± 3.64 (0.5–11.00)	3.18 ± 3.34 (0.00–10.50)	3.41 ± 2.03 (1.00–8.00)

Values are reported as means ± standard deviations (min–max). The threshold of statistical significance was set at *P* < 0.05. *P*-values refer to ANOVA models followed by *post hoc*, Bonferroni-corrected comparisons or Pearson’s χ^2^, as appropriate. bvFTD = behavioural variant frontotemporal dementia; CDR = clinical dementia rating; CDR plus NACC FTLD = Clinical Dementia Rating Dementia Staging Instrument plus National Alzheimer's Coordinating Center Behavior and Language Domains; CDR-sb = clinical dementia rating sum of boxes; MMSE = mini-mental state examination; nfvPPA = non-fluent/agrammatic variant primary progressive aphasia; sbvFTD = semantic behavioural variant frontotemporal dementia; svPPA = semantic variant primary progressive aphasia.

### Clinical and cognitive assessment

Clinical evaluation was performed as part of the diagnostic process by experienced neurologists, who recorded the disease duration at presentation. Global disease severity was assessed using the clinical dementia rating (CDR)^26^ and CDR Dementia Staging Instrument plus National Alzheimer's Coordinating Center Behavior and Language Domains (CDR plus NACC FTLD) scales.^27^ Participants underwent a comprehensive neuropsychological assessment (Supplementary Table 4), as previously described.^20^

### Genetic testing

The presence of pathological *C9orf72* expansions and/or known pathogenic mutations in *GRN*, *MAPT*, *FUS, TARDBP*, *TBK1*, *TREM2*, *OPTN* and *VCP* was assessed from blood samples using optimized protocols, as recently described.^28^ Mutation carriers were excluded from the present study.

### MRI acquisition

All participants of the study underwent brain MRI on a 3 T scanner (Philips Medical Systems). Details of MRI acquisition protocols [including T_2_-weighted, 3D fluid-attenuated inversion recovery (FLAIR), 3D high-resolution T_1_-weighted sequence and axial pulsed-gradient spin echo (PGSE) single shot diffusion-weighted (DW) EPI sequence] are provided in Supplementary Table 5.

### MRI analysis

MRI analysis was performed by experienced observers, blinded to the identity of subjects.

#### Diffusion-weighted MRI preprocessing

Preprocessing of DW imaging included correction for off-resonance and eddy current-induced distortions, and for movement, outlier detection and replacement using the Eddy tool within the FSL library. The process is described in the Supplementary materials. The diffusion tensor (DT) was estimated by linear regression using a multishell approach (three shells, with b = 700, 1000 and 2855 s/mm^2^), using the dtifit tool implemented in FSL. Subsequently, fractional anisotropy (FA) maps were derived. For the neurite orientation dispersion and density imaging (NODDI) model, intracellular volume fraction (ICVF) maps were computed using the NODDI Matlab Toolbox with default settings (http://www.nitrc.org/projects/noddi_toolbox).

#### Brain parcellation

The nodes of the brain network were identified using anatomical T_1_-weighted images. Grey matter was parcellated using a method based on 220 similarly sized areas, which combines the requirement for a large number of equally sized nodes with respecting anatomical landmarks.^29,30^ The 220 regions included the cerebral cortex and basal ganglia, and the cerebellum was excluded. The 220 grey matter regions of interest were moved into the subject’s space by calculating and concatenating the registrations between the subject’s T_1_-weighted image and MNI152 standard space [linear and non-linear using FLIRT^30^ and FNIRT,^31^ respectively, as implemented in FSL (FSL v.5.0.9; http://www.fmrib.ox.ac.uk/fsl)], and between the subject’s DT MRI (B0 image) and T_2_-weighted images (linear and non-linear, using FLIRT and FNIRT).

#### Network diffusion model

The NDM^8^ was implemented to simulate the hypothetical spread of disease-causing proteinopathy into the network represented by the connectivity matrix (*C*) over time, starting the diffusion process from a ‘seed’ region. Specifically, the diffusion model starts from baseline MRI data of the connectome of young healthy subjects *C* = (*e*, *n*), where *e_i_*_,*j*_ represents the pathways connecting structures *i* and *j*; *n_i_* represents the *i*th cortical or subcortical structure. The FA and ICVF measures were used to implement the model. The rationale behind this approach lies in the notion that the ‘healthy connectome’ might serve as a template for understanding the spread of pathological proteins and the consequent disruption of brain networks.^32^ Using young controls allows us to focus on the fundamental aspects of diffusion without the additional confounding introduced by ageing or pathology.

The spread of pathology from an affected brain region (R_2_) to an unaffected one (R_1_) is given by:


(1) $$\frac{dx_{1}}{dt}=\beta c_{1,2}(x_{2}-x_{1})$$

where *x*_1,2_ is the pathology concentration in region R_1,2_; *c*_1,2_ is the connectivity between R_1_ and R_2_; and β is the diffusivity constant (the higher the value, the higher is the speed of pathology progression).

Pathology from all brain regions is combined into a vector *x*(*t*)={*x_i_*(*t*)}*w*, and Equation (1) becomes:


(2) $$\frac{dx(t)}{dt}=-\beta Lx(t)$$

where *x*(*t*)={*x_i_*(*t*)}: represents the amount of diffusion of pathology at node *i* and time point *t* starting from an initial distribution at time *t* = 0(*x*(0)); *L* is the graph Laplacian matrix; and *t* are the time points (in arbitrary units).

From matrix algebra, Equation (1) is satisfied by:


(3) $$x(t)=e^{-\beta tL}x(0)$$

where *x*(0) is a vector with 1 at the index corresponding seed brain regions where it is thought that the pathology begins to spread, 0 at all other brain regions. Four disease epicentres were identified from the peaks of atrophy of each FTD variant: left temporal pole (svPPA), left insula (bvFTD), right temporal pole (sbvFTD) and left supplementary motor area (nfvPPA), as previously shown.^33,34^

The graph Laplacian represents the discretization of the Laplacian operator and indicates how a graph differs at one vertex from its values at nearby vertices. It was implemented as follows:


(4) $$L=I\;-D^{-\frac{1}{2}}C$$

where *I* is the identity matrix; *D* is the diagonal matrix whose diagonal entries contain the degree of each node; and *C* is the averaged connectome of healthy subjects.

Given that brain regions are not the same size, *L* in Equation (3) is the normalized version of the graph Laplacian operator. It is a symmetric matrix, and its eigenvectors are orthonormal. The solution of Equation (1) was implemented mathematically in Matlab by the eigenvalue decomposition:


(5) $$x(t)=\sum_{i=1}^{N}(e^{-\beta \lambda_{i}t}u_{i}^{t}x_{0})u_{i}$$

where *N* are the brain regions; *λ* are the eigenvalues of matrix *L*; and *u* are the eigenvectors of matrix *L*.

Brain Net Viewer was used to create diffusion maps^35^ (http://www.nitrc.org/projects/bnv/).

### Statistical analysis

#### Clinical and cognitive data

Demographic, clinical and cognitive/behavioural data were compared among FTD groups. ANOVA with a *post hoc* test was used for continuous variables (correcting *P*-values for multiple comparisons using the Bonferroni method) and the χ^2^ test for categorical variables. A two-sided *P*-value of <0.05 was considered for statistical significance. Statistical analysis was performed using the software R.

#### Correlation analysis

Network vulnerability was tested through correlation between predicted atrophy obtained by the NDM in young controls and the longitudinal pattern of atrophy in FTD patients. Specifically, Pearson’s correlation was calculated between the atrophy estimated by the NDM in young controls and the normalized atrophy obtained through the *t*-score, computed using Welch’s *t*-test as follows:^36^


(6) $$t=\frac{\mu_{\mathrm{HC}}-\mu_{\mathrm{pat}}}{\sqrt{\frac{\sigma_{\mathrm{HC}}^{2}}{N_{\mathrm{HC}}}+\frac{\sigma_{\mathrm{pat}}^{2}}{N_{\mathrm{pat}}}}}$$

where $\mu_{\mathrm{HC}}$ is the mean of the 220 grey matter volumes of healthy controls at baseline; $\mu_{\mathrm{pat}}$ is the mean of the patients’ volumes at the desired time point; $\sigma_{\mathrm{HC}}$ is the standard deviation (SD) of the healthy controls’ volumes at baseline; $\sigma_{\mathrm{pat}}$ is the SD of the patients’ volumes at the desired time point; $N_{\mathrm{HC}}$ is the number of healthy subjects; and $N_{\mathrm{pat}}$ is the number of patients.

#### Repeated seeding

The NDM approach was then run for all the 220 seed regions of interest to verify whether the regions chosen as seed were among those that yielded a high correlation. FA and ICVF measures were used separately to implement the model for each seed region. This process was repeated for each region, and the NDM-predicted pathology pattern was calculated. Pearson’s correlation coefficient was computed between each predicted pathology vector *x_i_*(*t*) seeded at region *i* and the empirical pathology vector Φ across all model time points *t*, yielding *R_i_*(*t*). The maximum value of *R_i_*(*t*), denoted as *R_i_*_,max_, was recorded for each region. A histogram of the maximum *R* achieved from all regions of interest seeded from each phenotype was obtained. For each histogram, a significant cut-off of 1.96 SDs (σ) in the upper bound of the tail of the null hypothesis distribution was identified. We then checked whether the four disease epicentres, identified based on the peaks of atrophy for each FTD phenotype, had an *R_i_*_,max_ value of >1.96σ.

## Results

### Clinical and cognitive features

All FTD groups were comparable in terms of education, disease duration, CDR, CDR plus NACC FTLD and CDR sum of boxes scores (Table 1). Patients with bvFTD showed additional impairment of visuospatial abilities and worse executive performance compared with svPPA. svPPA patients showed significant impairment of confrontation naming, single-word comprehension and semantic knowledge, compared with nfvPPA (Supplementary Table 3).

### Network diffusion model

**Figure 1:**
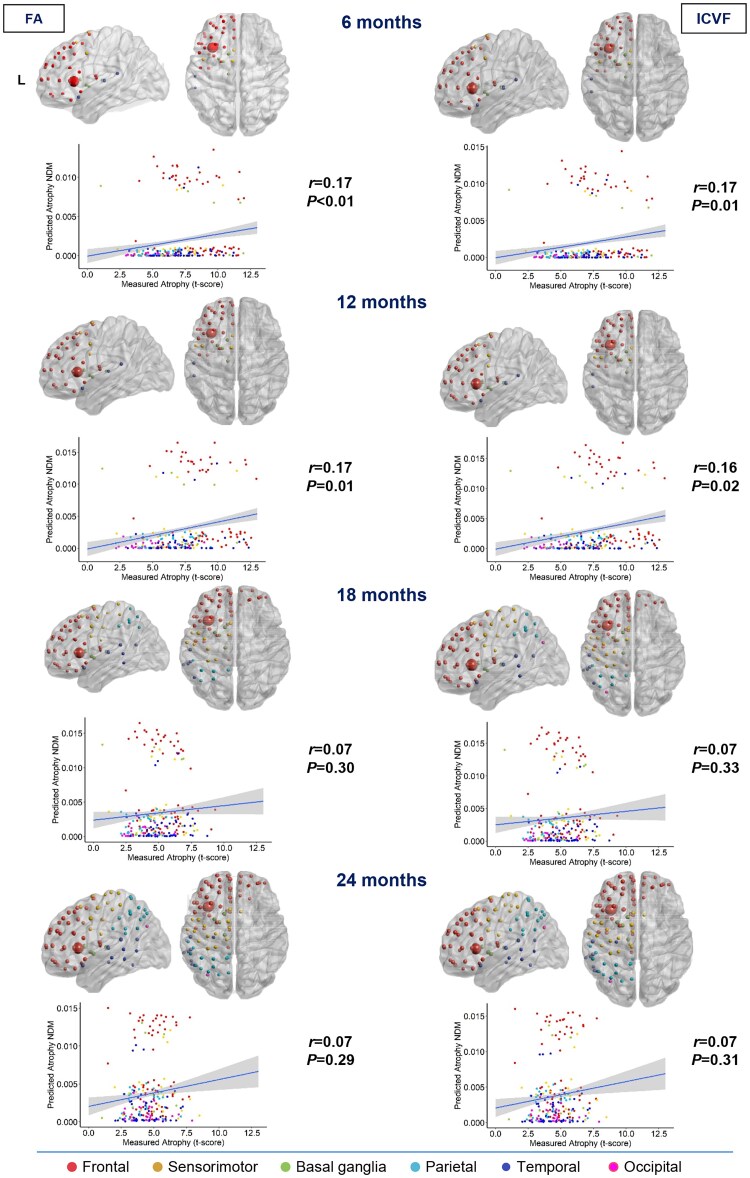
**Spatial distribution of behavioural variant frontotemporal dementia estimated atrophy predicted by a network diffusion model applied to the fractional anisotropy connectome and intracellular volume fraction connectome starting from the left insula.** The results are visualized in sagittal and axial views using a ‘glass brain’ representation, where spheres are placed at the centroid of each brain region. The diameter of each sphere is proportional to the effect size of atrophy, and spheres are colour-coded by lobe. Additionally, correlations between normalized atrophy, as measured by *t*-scores in behavioural variant frontotemporal dementia patients, and atrophy estimated by the network diffusion model (NDM) in young controls are plotted on scatter plots. These correlations are based on fractional anisotropy (FA) and intracellular volume fraction (ICVF) matrices at different time points (6, 12, 18 and 24 months).

**Figure 2:**
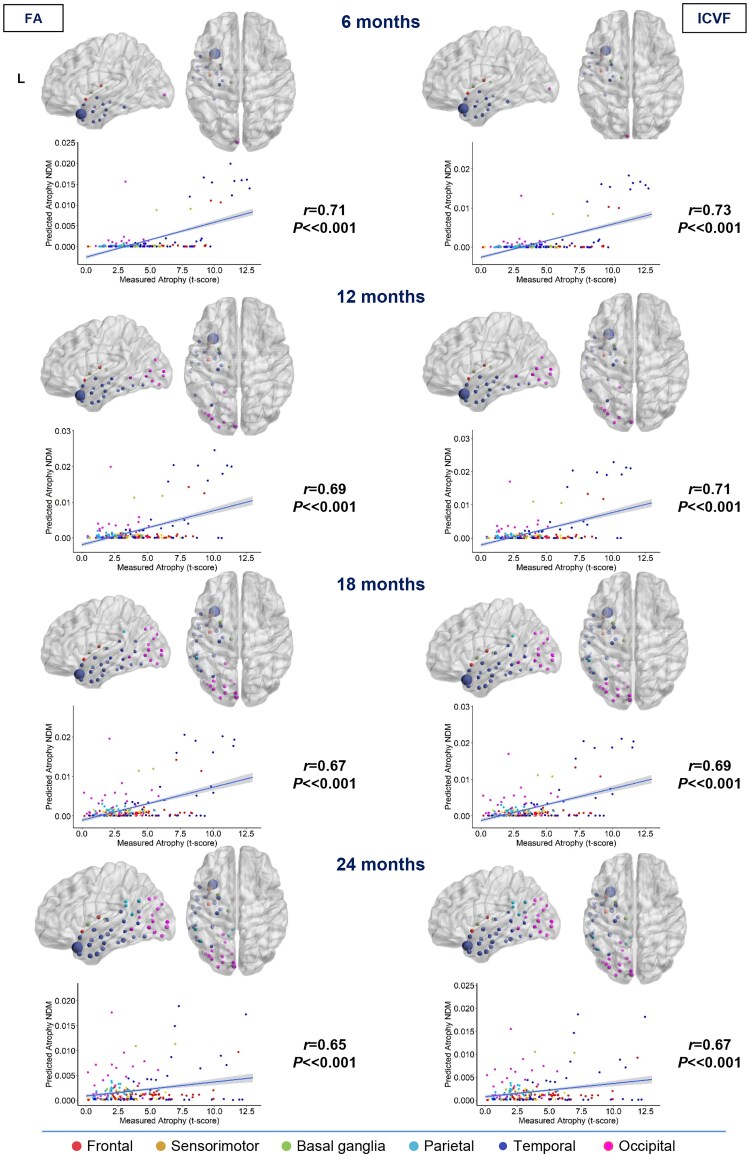
**Spatial distribution of semantic variant primary progressive aphasia estimated atrophy predicted by a network diffusion model applied to the fractional anisotropy connectome and intracellular volume fraction connectome.** The results are visualized in sagittal and axial views using a ‘glass brain’ representation, where spheres are placed at the centroid of each brain region. The diameter of each sphere is proportional to the effect size of atrophy, and spheres are colour-coded by lobe. Additionally, correlations between normalized atrophy, as measured by *t*-scores in semantic variant primary progressive aphasia patients, and atrophy estimated by the network diffusion model (NDM) in young controls are plotted on scatter plots. These correlations are based on fractional anisotropy (FA) and intracellular volume fraction (ICVF) matrices at different time points (6, 12, 18 and 24 months).

**Figure 3:**
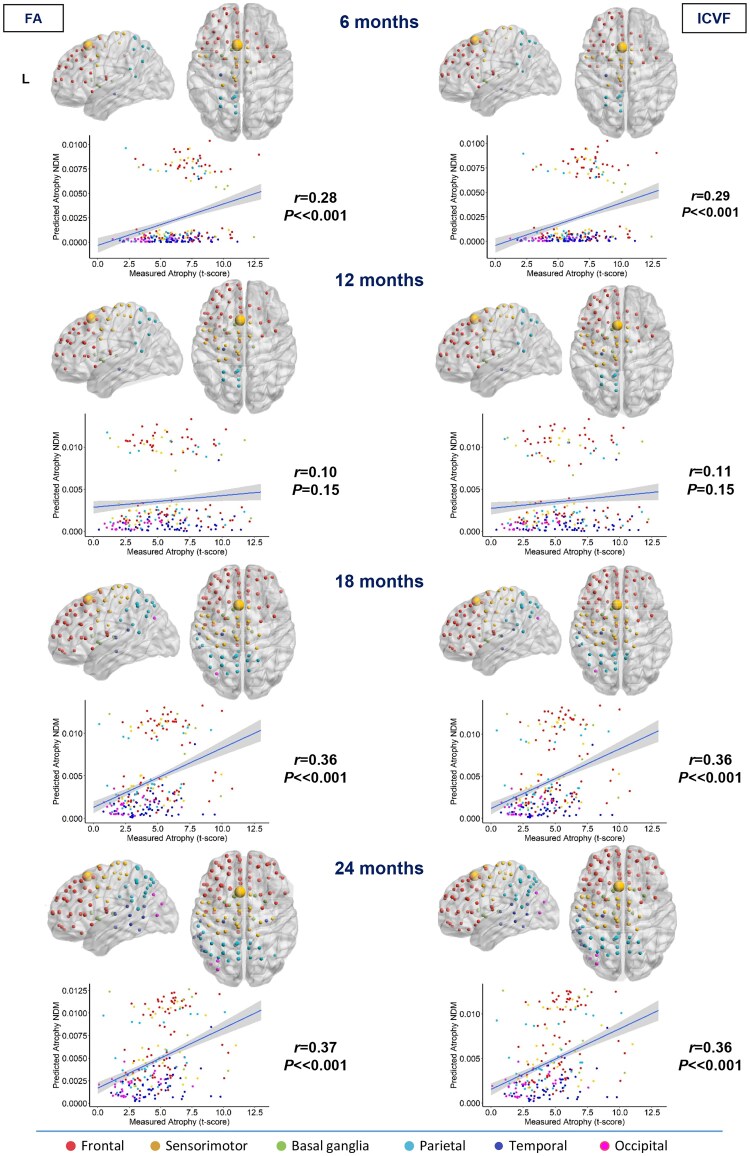
**Spatial distribution of non-fluent/agrammatic variant primary progressive aphasia estimated atrophy predicted by a network diffusion model applied to the fractional anisotropy connectome and intracellular volume fraction connectome.** The results are visualized in sagittal and axial views using a ‘glass brain’ representation, where spheres are placed at the centroid of each brain region. The diameter of each sphere is proportional to the effect size of atrophy, and spheres are colour-coded by lobe. Additionally, correlations between normalized atrophy, as measured by *t*-scores in non-fluent/agrammatic variant primary progressive aphasia patients, and atrophy estimated by the network diffusion model (NDM) in young controls are plotted on scatter plots. These correlations are based on fractional anisotropy (FA) and intracellular volume fraction (ICVF) matrices at different time points (6, 12, 18 and 24 months).

**Figure 4:**
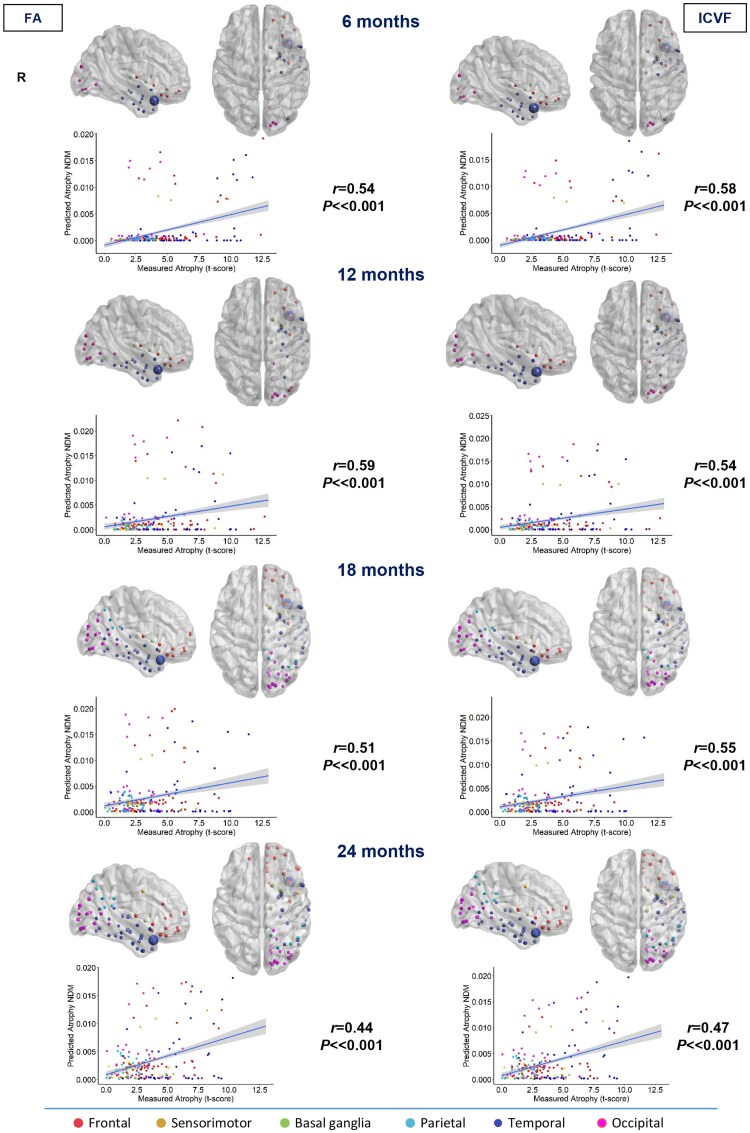
**Spatial distribution of semantic behavioural variant frontotemporal dementia estimated atrophy predicted by a network diffusion model applied to the fractional anisotropy connectome and intracellular volume fraction connectome.** The results are visualized in sagittal and axial views using a ‘glass brain’ representation, where spheres are placed at the centroid of each brain region. The diameter of each sphere is proportional to the effect size of atrophy, and spheres are colour-coded by lobe. Additionally, correlations between normalized atrophy, as measured by *t*-scores in semantic behavioural variant frontotemporal dementia patients, and atrophy estimated by the network diffusion model (NDM) in young controls are plotted on scatter plots. These correlations are based on fractional anisotropy (FA) and intracellular volume fraction (ICVF) matrices at different time points (6, 12, 18 and 24 months).

The predictive maps obtained by NDM in young controls are reported in Figs 1–4, where the progression of pathology from each seed, at each time point, is represented. The biggest node (proportional to the regional atrophy) is in the region chosen as seed, whereas the other nodes represent how much and where the pathology is more likely to spread.

In the case of bvFTD, considering both FA and ICVF measures, NDM predicted an early spread from the left insular seed to many frontal regions, sensorimotor (precentral gyrus and supplementary motor area) and superior temporal lobes, and to the basal ganglia (caudate, putamen, pallidum and thalamus) in the left hemisphere (6 and 12 months). After 18 months, the left parietal lobe is also involved. Between 18 and 24 months, there is evident involvement of the frontal areas of the contralateral hemisphere (right). The left part of the occipital lobe was found to be affected at the last follow-up (24 months) when considering FA measures and at 18 months with ICVF (Fig. 1).

In svPPA, considering FA and ICVF measures, the left temporal lobe, basal ganglia (6 months) and occipital lobe (12 months) are reached early by pathology according to the the NDM. At 18 months, the left parietal lobe (supramarginal gyrus) has become involved. At the last time point (24 months), the disease has started to affect most of the left hemisphere in the frontal, temporal and occipital lobes. Maps constructed with FA and ICVF measures suggested a similar spreading of the disease (Fig. 2).

The FA and ICVF maps suggested an early spread of nfvPPA pathology to the bilateral frontal and sensorimotor (supplementary motor area and postcentral gyrus), left parietal and temporal (hippocampus) regions and basal ganglia (left caudate, putamen and pallidum, and bilateral thalamus) (6 and 12 months). At 18 months, the left middle occipital lobe has been reached. At the last time point (24 months), many regions in the right hemisphere, including frontal and parietal lobes, have also become involved. The right hippocampus (temporal lobe) was found to be affected at the last follow-up (24 months) only in the maps created with FA measures (Fig. 3).

Taking into account FA and ICVF measures, maps suggested a spread of sbvFTD pathology to the right hemisphere including the temporal, frontal and occipital (superior and inferior occipital gyri, calcarine fissure and lingual gyrus) lobes and basal ganglia (caudate, putamen and thalamus) (6 and 12 months). At 18 months, parietal lobar regions have been reached, involving the right angular gyrus and precuneus. At the last time point (24 months), many regions of the right hemisphere in the temporal, parietal and occipital lobes have become involved, and there is initial alteration of the sensorimotor areas (precentral gyrus). Maps constructed with FA and ICVF measures suggested a similar spreading of the disease (Fig. 4).

### NDM-predicted versus real atrophy

The degree of atrophy predicted in each region by the NDM applied on the FA and ICVF connectome in healthy young subjects showed moderate but significant positive correlation with the observed longitudinal pattern in bvFTD patients at 6 and 12 months (*r* range, 0.16–0.17; *P*-value range, 0.01–0.02), whereas no significant correlations were found at further time points (Fig. 1). In contrast, the degree of predicted atrophy showed a strongly significant positive correlation with the longitudinal pattern of patient atrophy observed in sbvFTD (*r* range, 0.44–0.59; *P* < 0.001) and in svPPA patients (*r* range, 0.65–0.73; *P* < 0.001) at all time points (6, 12, 18 and 24 months), as shown in Figs 2 and 4. Finally, nfvPPA patients showed a significant correlation at 6, 18 and 24 months (*r* range, 0.28–0.37; *P* < 0.001; Fig. 3).

Overall, pathology diffusion predicted by NDM applied on the ICVF connectome (*r* range, 0.16–0.73; *P*-value range, <0.001–0.01) from disease epicentres of each FTD variant demonstrated slightly higher values of correlation compared with atrophy predicted by NDM applied on FA maps (*r* range, 0.17–0.71; *P*-value range, <0.001–0.02).

### Repeated seeding

**Figure 5:**
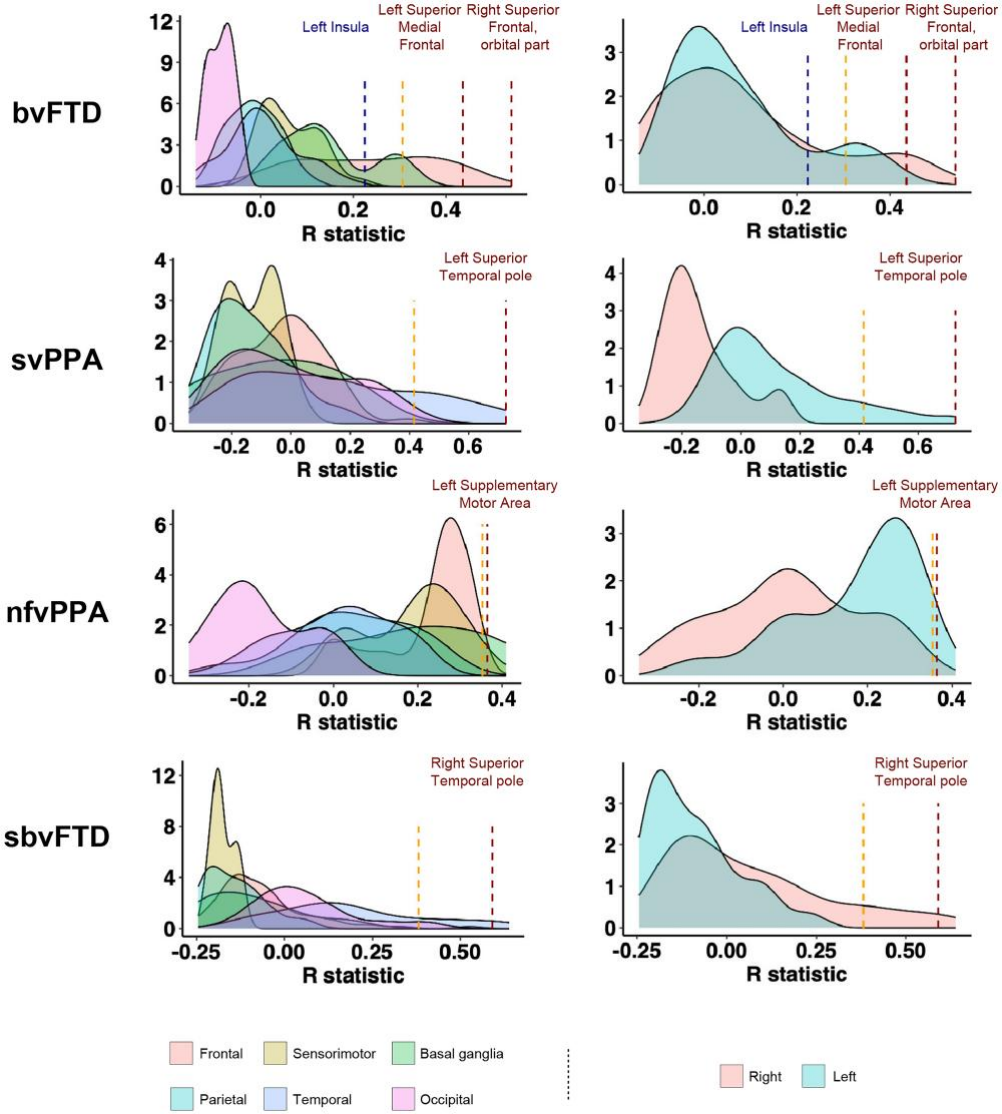
**Histogram of the maximum *R* achieved from all regions of interest seeded from each phenotype using the intracellular volume fraction measure of the structural connectome.** The figure is divided into two parts: the *left* represents histograms with brain hemispheres, each shown in a different colour, and the *right* represents different brain lobes, also displayed in distinct colours. For each histogram, a significant cut-off at 1.96 SD (σ) in the upper bound of the tail of the null hypothesis distribution is identified (indicated by dashed orange bars). Previously identified disease epicentres, located at the peaks of atrophy in behavioural variant frontotemporal dementia (bvFTD, left and right superior medial frontal cortex), semantic variant primary progressive aphasia (svPPA, left temporal pole), semantic behavioural variant FTD (sbvFTD, right temporal pole) and non-fluent/agrammatic variant PPA (nfvPPA, left supplementary motor area), are marked with dashed dark red lines.

**Figure 6:**
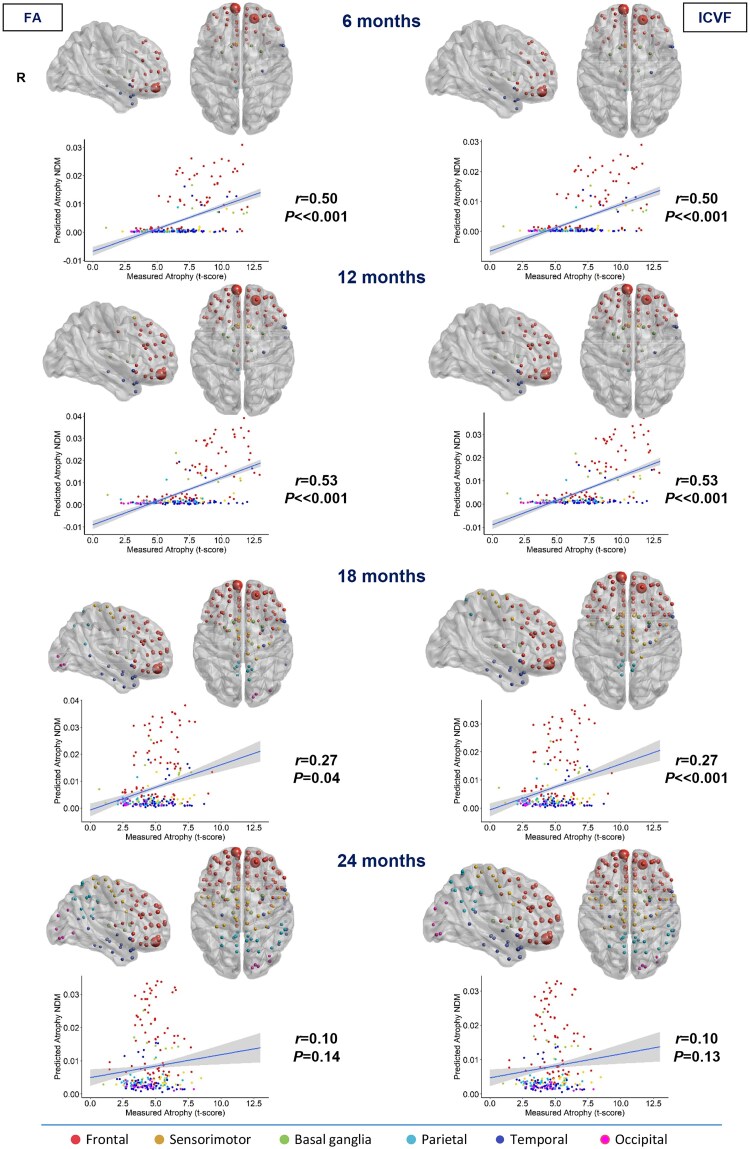
**Spatial distribution of behavioural variant frontotemporal dementia estimated atrophy predicted by a network diffusion model applied to the fractional anisotropy connectome and intracellular volume fraction connectome starting from the right superior frontal gyrus (orbital part) and left superior medial frontal cortex.** The results are visualized in sagittal and axial views using a ‘glass brain’ representation, where spheres are placed at the centroid of each brain region. The diameter of each sphere is proportional to the effect size of atrophy, and spheres are colour-coded by lobe. Additionally, correlations between normalized atrophy, as measured by *t*-scores in behavioural variant frontotemporal dementia patients, and atrophy estimated by the network diffusion model (NDM) in young controls are plotted on scatter plots. These correlations are based on fractional anisotropy (FA) and intracellular volume fraction (ICVF) matrices at different time points (6, 12, 18 and 24 months).

Each region was computationally ‘seeded’ in turn, and NDM was run over time. A histogram of the maximum *r* achieved from all regions of interest seeded from each phenotype was obtained (Fig. 5 for ICVF and Supplementary Fig. 1 for FA). Each colour represents different brain lobes or brain hemispheres. For each histogram, a significant cut-off of 1.96σ in the upper bound of the tail of the null hypothesis distribution was identified (dashed red bars in the histogram). The previously identified disease epicentres, located at the peaks of atrophy in svPPA (left temporal pole), in sbvFTD (right temporal pole) and in nfvPPA (left supplementary motor area), were found with an *r_i_*_,max_ of >1.96σ (dashed orange bars in the histogram; for the list of regions, see Supplementary Tables 6 and 7). Regions with a high correlation are all located in the left hemisphere for svPPA and nfvPPA and in the right hemisphere for sbvFTD. Conversely, the peak for bvFTD patients, located in the left insula, does not fall within this range. Notably, bvFTD is the only variant to have regions in both the right and left hemispheres with an *r_i_*_,max_ of >1.96σ. Therefore, for this phenotype, we selected the highest-ranking region in each hemisphere: the right superior frontal gyrus (orbital part) and the left superior medial frontal cortex (Fig. 6). These two regions are also among the most atrophic in bvFTD, as previously demonstrated.^33,34^ In bvFTD, considering both FA and ICVF measures, the NDM predicted an early spread from the bilateral superior frontal seeds to many frontal regions, sensorimotor (supplementary motor area) and superior and middle temporal lobes, and to the basal ganglia (caudate, putamen, pallidum and thalamus) in the left and right hemisphere (6 and 12 months). After 18 months, the left and right sensorimotor (precentral, paracentral and postcentral gyri) and parietal lobes (precuneus) are also involved. The right part of the occipital lobe (calcarine, lingual and superior and middle occipital gyri) was found to be affected at 18 and 24 months when considering FA measures and at 24 months with ICVF (Fig. 6). The degree of atrophy predicted in each region by NDM applied on the FA and ICVF connectome in healthy young subjects showed moderate but significant positive correlation with the observed longitudinal pattern in bvFTD patients at 6, 12 and 18 months (*r* range, 0.27–0.53; *P*-value range, <0.001–0.04), whereas no significant correlations were found at 24 months (Fig. 6).

## Discussion

The focus of the present MRI study was on investigating the applications of an NDM based on structural connectomic data to assess whether the progression of FTD pathology over time might be modelled by a network-based spreading process from a disease epicentre. We showed that NDM-predicted patterns of atrophy displayed a significant correspondence to the real evolution of atrophy observed longitudinally across different clinical variants of the FTD spectrum of disorders, including the poorly characterized sbvFTD. These findings are consistent with the view that misfolded proteins spread through highly interconnected vulnerable brain regions.^7,37^ Of note, the highest correlation coefficients were found for the most focal and pathologically homogeneous variants, such as svPPA and sbvFTD, which are mostly associated with a TDP-43 proteinopathy. We also compared structural metrics obtained from a ‘classic’ DTI model (i.e. FA) with those provided by NODDI (in particular, ICVF), demonstrating similar results.

In a previous cross-sectional study,^33^ we tested whether the severity of regional atrophy across FTD clinical variants was correlated with the topological distance from the respective disease epicentres, as described by functional connectomic data. Using this approach, although we did show a significant correlation between the atrophy patterns of svPPA patients and the topological distance from a region of interest placed in the left inferior temporal gyrus, such correlation did not prove significant for patients with bvFTD and nfvPPA. Moreover, that study lacked a validation of the predictive value of network-based metrics for progression of pathology over time, which can be obtained only with a longitudinal study design. In addition, the evaluation of functional MRI data did not allow strong conclusions regarding the physical progression of pathological proteins through synaptic connections. In the present study, moving from observations drawn by investigators in an independent cohort of bvFTD patients,^8^ we aimed to overcome such shortcomings by applying an NDM based on brain structural connectomic data to a longitudinal cohort of patients presenting with several clinical variants of the FTD spectrum. The inclusion of a sizeable group of patients with sbvFTD holds particular interest, considering the recent efforts for its definition as a specific clinicopathological entity, to be distinguished from bvFTD and svPPA.^19,34,38^

In the case of bvFTD, predictive maps showed a pattern of spread of pathology consistent with the four neuropathological stages of TDP-43 pathology defined by Brettschneider *et al*.^3^ and Kassubek *et al*.^39^ In detail, stages I and II are characterized by pathological accumulation in the orbital gyri, gyrus rectus, inferior frontal, middle frontal and superior frontal and superior temporal gyri, in addition to subcortical structures including the striatum and thalamus, similar to what we observed in the predictive maps at the 6- and 12-month time points. Pathological stage III involves the motor and parietal cortices, whereas stage IV also involves the visual cortex, similar to our findings based on NDM predictions at the 18- and 24-month time points, respectively. However, when the NDM started from the peak of atrophy (namely the left insula), a significant correlation between predicted and observed atrophy was detected only at 6 and 12 months, with coefficients indicating an overall weak correlation. In addition, the NDM maintained a strongly lateralized (i.e. left hemispheric) pattern of atrophy even at later time points, which is not consistent with the bilateral pattern of atrophy that was observed in our bvFTD cohort (as described by Ghirelli *et al*.^34^). In bvFTD, we found that the progression of atrophy was much better explained by using the bilateral superior frontal gyrus as the model seeds, which showed the highest correlation ranking between the real and the predicted atrophy in both the right and left hemispheres. Among the possible explanations for these findings, we suggest that the intrinsic neuropathological heterogeneity of bvFTD might play an important role, because this clinical variant is known to be caused almost equally by either FTLD-tau or FTLD-TDP pathology.^40^ As a matter of fact, in the current absence of a pathological staging of tau pathology in FTLD or any definite *in vivo* biomarker to distinguish between the two main FTLD pathologies,^41^ there is still uncertainty regarding divergence in the location of disease epicentres and ‘target’ networks according to the underlying proteinopathy in patients with bvFTD. Therefore, in our cohort lacking post-mortem neuropathological confirmation, we could not discriminate between these two different sub-populations. Models accounting for individual differences in epicentre location could improve prediction accuracy in this phenotype.

In contrast, the NDM starting from the peaks of atrophy performed very well in FTD variants with a more strongly lateralized and consistent disease epicentre across patients, such as svPPA, sbvFTD and nfvPPA. In these cases, the model accurately captured the asymmetric spread of pathology. Compared with bvFTD, we showed a much greater correspondence between NDM-predicted and empirically observed patterns of atrophy in both sbvFTD and svPPA, which are known to harbour a TDP-43 neuropathology in the majority of cases.^42,43^ To our knowledge, there is no previous evidence regarding the longitudinal evolution of brain atrophy of the sbvFTD variants (also known as right temporal variant of FTD), which has been characterized in its diagnostic features only recently^19^ and has mostly been considered as a ‘mirror’ variant of svPPA.^43^ Previous studies in svPPA patients showed that the earliest changes include left hemispheric prevalent grey matter loss in the inferior temporal and fusiform gyri, followed by an involvement of the posterior temporal regions, inferior parietal lobule and occipital lobe, consistent with progression of pathology through axonal connections in the inferior longitudinal fasciculus.^44,45^ Although the left hemisphere is typically much more involved than the contralateral, significant involvement of the right anterior temporal regions is also reported in svPPA. Consistent with previous knowledge, our NDM maps showed a similar pattern of spreading through the left hemisphere in svPPA that was strongly correlated with the real pattern of atrophy in our patients, although we did not observe a contralateral spread to the right hemisphere. Our present finding is also in line with a previous cross-sectional study by our group using functional MRI data,^33^ providing further evidence supporting the view that healthy brain architecture from the disease epicentre shapes the pattern of distribution of atrophy in svPPA, and additionally proving that it can be used to predict its evolution longitudinally over time. In sbvFTD, NDM prediction of pathology spread from the right temporal pole (i.e. a perfectly specular right hemispheric representation of the svPPA epicentre) was mostly consistent with the pattern of svPPA. However, an additional involvement of the frontal regions was predicted very early according to the NDM (i.e. from the 6-month time point), suggesting that the target network of this variant might be more widespread than svPPA. Such evidence supports the current efforts to define sbvFTD as a separate entity specifically affecting a right hemispheric socioemotional semantic network^19,46,47^ and not a simple ‘mirror’ of svPPA.

In nfvPPA patients, NDM prediction of spreading from the left supplementary motor area showed an early progression involving the left frontal operculum, premotor area, anterior insula, superior parietal gyrus, precuneus and striatal regions, consistent with a pathological progression through the frontal aslant tract, superior longitudinal fasciculus and fronto-striatal tracts.^44,48^ nfvPPA predictive maps showed an initial lateralized spread, in particular to the left frontal, superior temporal and inferior parietal lobes, with a later involvement of contralateral areas, consistent with previous reports in the literature.^49,50^ Also for this variant, the significant correlations that we found with the empirical longitudinal atrophy patterns at most of the time points (i.e. 6, 18 and 24 months) support the hypothesis that the healthy architecture of the structural connectome might influence the spatiotemporal progression of atrophy and underlying pathology, which is, in most cases, a tauopathy.^42^

The accuracy values of each model showed that ICVF offered a slightly greater specificity than FA to model pathology spread, probably owing to the greater ability of the NODDI model to delineate the overall structural architecture of the brain. This becomes particularly important in the FTD spectrum, where the pathology involves not only axonal loss but also complex changes in white matter integrity, such as demyelination and alterations in fibre orientation.^51^ The ability of NODDI to delineate these variations is crucial, especially in regions with a high presence of crossing fibres, where FA might lack precision owing to its sensitivity to overall diffusion directionality.^51^ However, such an effect was only mild, and applying this technique in larger samples will be crucial to replicate our results and validate NDM based on the NODDI model as the ‘gold standard’.

The field of network connectivity and predictive models is evolving rapidly. The NDM uses a mathematical framework based on passive diffusion to describe how pathology propagates along anatomical brain connections. By using the graph Laplacian derived from structural or functional connectivity, the model effectively predicts disease progression on a macroscopic scale. However, it does not account for patient-specific variables or genetic factors, which can be relevant to FTD. In contrast, models based on individualized epicentres^17^ predicted atrophy in FTD by identifying an initial disease epicentre and modelling pathological spread along functional connections. These models incorporate metrics such as shortest path length and nodal hazard, making them more adaptable to individual cases. More recently, models incorporating transcriptional vulnerability have emphasized the role of genetic differences in determining why specific brain regions experience greater atrophy in bvFTD.^52^ This approach reveals how inherent genetic vulnerabilities contribute to regional susceptibility. Finally, advanced models, such as the epidemic spreading model, expand upon the NDM by integrating local biological processes such as protein production, aggregation and clearance, enabling more accurate predictions across various neurodegenerative proteinopathies.^53^

This study is not without limitations. Firstly, a significant limitation of this study is the lack of pathological confirmation of FTLD diagnoses; although, when available, CSF results were not suggestive for AD pathology. Secondly, the *a priori* definition of the epicentre in NDM (i.e. the most atrophic region) is only one of the possible approaches and, as such, might have limited the generalizability of the conclusions. NDM seeding could ideally be repeated from every node, and the one giving the highest correlation coefficient could be selected as the seed. A possible alternative is also to do this on individual subjects and show the ‘seed likelihood map’ across all subjects. Third, the sample is relatively small. The rarity of these conditions and the difficulty in obtaining longitudinal MRI scans make it particularly challenging to obtain a comprehensive dataset. This limitation underscores the need for collaborative efforts and larger multi-centre studies to enhance the robustness and generalizability of findings in rare disease research. Moreover, the lack of a reference standard for the regional parcellation of brain MR imaging can markedly affect graph theoretical metrics, meaning that comparisons with previous MRI studies using different approaches can be challenging. Furthermore, the NDM is a first-order, linear model of diffuse spread that assumes that the structural connection network stays constant during the progression of the illness. Although all neurodegenerative disorders result in abnormal structural connections, constant connectomes, such as the ones used here, typically do not significantly reduce the predictive power of the model. Therefore, future studies aimed to gain a better understanding of the mechanism of atrophy spread in neurodegenerative conditions should also assess non-linear active modelling.

Despite these limitations, here we showed that the implementation of NDM to cross-sectional structural connectome data is a valuable tool to predict future patterns of atrophy and spreading of pathology in the main variants of the FTD spectrum, particularly for those variants with a more definite neuropathological underpinning (i.e. TDP-43 for svPPA/sbvFTD and tau for nfvPPA). Future studies on data obtained from independent cohorts of patients with a pathologically proven diagnosis of FTD will be crucial to validate our models and provide definite evidence for the network-based degeneration hypothesis in this heterogeneous group of neurodegenerative diseases. Moreover, future directions include incorporation of local transcriptomic vulnerability into the NDM, as previously demonstrated with other mathematical models in bvFTD.^52^ With a larger sample size, this approach will allow for better capturing of regional variability in disease spread influenced by genetic factors in all FTD phenotypes.

## Supplementary Material

awae391_Supplementary_Data

## Data Availability

The dataset used and analysed during the present study will be made available by the corresponding author upon request to qualified researchers (i.e. affiliated to a university or research institution/hospital).
