# Supplementary material for: Modelling pathological spread through the structural connectome in the frontotemporal dementia clinical spectrum
Source: Brain. 2024 Nov 29;148(6):1994–2007. doi: 10.1093/brain/awae391 (PMC12129736; doi:10.1093/brain/awae391)
Supplement: awae391_Supplementary_Data [file awae391_supplementary_data.pdf]

SUPPLEMENTARY MATERIALS

Neuropsychological assessment

The neuropsychological assessment was performed by experienced neuropsychologists, unaware of MRI results. The following domains were investigated: global cognitive functioning with the Mini Mental State Examination (MMSE)[1] and the frontal assessment battery (FAB);[2] long and short term verbal memory with the Rey Auditory Verbal Learning Test[3] and the digit span forward,[4] respectively; long and short term visuospatial memory with the recall of the Reys complex figure [5] and the spatial span forward;[4] attention and executive functions with the digit span backward,[6] Ravens colored progressive matrices (RCPM),[7] Trail Making Test,[8] attentive matrices[9] and the Modified Card Sorting Test;[10] social cognition with the intention and emotion attribution of the Story-based Empathy Task (SET),[11] and the subtests (affect discrimination, affect naming, affect matching and affect selection) of the Comprehensive Affect Testing System (CATS);[12] language with the token test,[13] and with the semantic and phonemic fluencies;[14] visuospatial abilities with the Clock Drawing Test (CDT),[15] and the copy of Reys complex figure[5] and of drawings without landmarks;[3] praxis was tested with orofacial ideomotor apraxia test,[9] and with the Goldenbergs test;[16] the presence of behavioral disturbances with the A and B sections of the frontal behavioral inventory (FBI)[17] administered to patients caregivers. The healthy controls were administered with the same neuropsychological assessment except for FAB, CDT, SET, orofacial and Goldenberg ideomotor apraxia, FBI.

For the machine learning analysis, the following neuropsychological variables were selected: FAB, RCPM, token test, phonemic and semantic fluency, digit span backward, SET (emotion attribution), FBI A and B.

Diffusion weighted MRI Pre-processing

Preprocessing of diffusion-weighted data included skull-stripping and correction for head motions by aligning the volumes to the first B0 volume, as well as susceptibility-induced field and eddy currents distortion correction, using the tools implemented in the FMRIB software library (FSL, version 5.0.9).

REFERENCES

1. Folstein MF, Folstein SE, McHugh PR. "Mini-mental state". A practical method for grading the cognitive state of patients for the clinician. *J Psychiatr Res*. Nov 1975;12(3):189-98. doi:10.1016/0022-3956(75)90026-6
2. Appollonio I, Leone M, Isella V, *et al*. The Frontal Assessment Battery (FAB): normative values in an Italian population sample. *Neurol Sci*. Jun 2005;26(2):108-16. doi:10.1007/s10072-005-0443-4
3. Carlesimo GA, Caltagirone C, Gainotti G. The Mental Deterioration Battery: normative data, diagnostic reliability and qualitative analyses of cognitive impairment. The Group for the Standardization of the Mental Deterioration Battery. *Eur Neurol*. 1996;36(6):378-84. doi:10.1159/000117297
4. Orsini A, Grossi D, Capitani E, Laiacona M, Papagno C, Vallar G. Verbal and spatial immediate memory span: normative data from 1355 adults and 1112 children. *Ital J Neurol Sci*. Dec 1987;8(6):539-48. doi:10.1007/BF02333660
5. Caffarra P, Vezzadini G, Dieci F, Zonato F, Venneri A. Rey-Osterrieth complex figure: normative values in an Italian population sample. *Neurol Sci*. Mar 2002;22(6):443-7. doi:10.1007/s100720200003
6. Monaco M, Costa A, Caltagirone C, Carlesimo GA. Forward and backward span for verbal and visuo-spatial data: standardization and normative data from an Italian adult population. *Neurol Sci*. May 2013;34(5):749-54. doi:10.1007/s10072-012-1130-x
7. Basso A, Capitani E, Laiacona M. Ravens coloured progressive matrices: normative values on 305 adult normal controls. *Funct Neurol*. Apr-Jun 1987;2(2):189-94.
8. Giovagnoli AR, Del Pesce M, Mascheroni S, Simoncelli M, Laiacona M, Capitani E. Trail making test: normative values from 287 normal adult controls. *Ital J Neurol Sci*. Aug 1996;17(4):305-9. doi:10.1007/BF01997792
9. Spinnler H, Tognoni, G. . Standardizzazione e taratura italiana di test neuropsicologici. *Ital J Neurol Sci* 1987;6(8):44-46.
10. Caffarra P, Vezzadini G, Dieci F, Zonato F, Venneri A. Modified Card Sorting Test: normative data. *J Clin Exp Neuropsychol*. Apr 2004;26(2):246-50. doi:10.1076/jcen.26.2.246.28087
11. Dodich A, Cerami C, Canessa N, *et al*. A novel task assessing intention and emotion attribution: Italian standardization and normative data of the Story-based Empathy Task. *Neurol Sci*. Oct 2015;36(10):1907-12. doi:10.1007/s10072-015-2281-3
12. Schaffer SG, Wisniewski A, Dahdah M, Froming KB. The comprehensive affect testing system-abbreviated: effects of age on performance. *Arch Clin Neuropsychol*. Feb 2009;24(1):89-104. doi:10.1093/arclin/acp012
13. De Renzi E, Vignolo LA. The token test: A sensitive test to detect receptive disturbances in aphasics. *Brain*. Dec 1962;85:665-78. doi:10.1093/brain/85.4.665
14. Novelli G LM, Papagno C, Vallar G, Capitani E, Cappa SF. . Three clinical tests to research and rate the lexical performance of normal subjects. . *Arch Psicol Neurol Psichiatr* 1986;47:477–506.
15. Manos PJ. Ten-point clock test sensitivity for Alzheimers disease in patients with MMSE scores greater than 23. *Int J Geriatr Psychiatry*. Jun 1999;14(6):454-8.
16. Goldenberg G. Neuropsychological assessment and treatment of disorders of voluntary movement.

. In: Halligan K, & Marshall ed. *Handbook of clinical neuropsychology* 2003.
17. Alberici A, Geroldi C, Cotelli M, *et al*. The Frontal Behavioural Inventory (Italian version) differentiates frontotemporal lobar degeneration variants from Alzheimers disease. *Neurol Sci*. Apr 2007;28(2):80-6. doi:10.1007/s10072-007-0791-3

Supplementary Table 1. Number of longitudinal follow-up MRI scans for each subject group.

	**bvFTD**	**svPPA**	**nfvPPA**	**sbvFTD**
**Follow-up 6 months**	25	11	12	10
**Follow-up 12 months**	17	7	6	7
**Follow-up 18 months**	12	7	7	6
**Follow-up 24 months**	10	6	7	5

Abbreviations: bvFTD= behavioral variant frontotemporal dementia; nfvPPA= nonfluent/agrammatic variant primary progressive aphasia; sbvFTD= semantic behavioral variant frontotemporal dementia;svPPA= semantic variant primary progressive aphasia.

Supplementary Table 2. Demographic features of young healthy controls.

	**N**	**Age at MRI [years]**	**SEX [men/ women]**	**Education [years]**
**YOUNG CONTROLS**	48	24.67 ± 2.74 (20.19- 30.66)	26/22	15.38 ± 2.78 (8.00-21.00)

Values are means ± standard deviations [range].

Supplementary Table 3. Neuropsychological features of young healthy controls.

	**Test**	**Mean ± SD (range)**
**Global cognition**	**MMSE**	29.82±0.39 (29.00-30.00)
**Executive function**	**PASAT, two seconds**	40.64±9.97 (18.00-58.00)
	**MCST, categories**	5.49±0.87 (3.00-6.00)
	**TMT, B-A**	36.45±14.72 (14.01-105.40)
**Memory**	**RAVLT, delayed recall**	12.77±1.80 (8.00-15.00)
	**Reys complex figure, recall**	23.56±4.72 (11.00-33.00)
**Language**	**CaGi, naming in response to an oral description**	47.09±1.21 (43.00-48.00)
**Visuospatial abilities**	**Reys complex figure copy**	32.89±2.10 (26.00- 36.00)
	**Benton, judgement of line orientation**	27.36±2.64 (18.00-30.00)
**Mood & behaviour**	**BDI**	5.87±4.85 (0.00-24.00)

Values are means ± standard deviations [range]. Abbreviations: BDI = Becks Depression Inventory; MCST = modified card sorting test; MMSE = Mini-mental-state evaluation; RAVLT=Rey Auditory Verbal Learning Test; TMT = Trail-making test

Supplementary Table 4. Neuropsychological features of the cohort.

Variables	bvFTD	svPPA	nfvPPA	sbvFTD
**Number**	35	20	14	9
Global cognition				
**MMSE**	23.57±5.54 (6.00-30.00)	21.58±7.62 (5.00-30.00)	24.17±7.82 (5.00-30.00)	24.87±4.61 (18.00-30.00)
**FAB**	11.32±4.01 (3.00-17.00)	11.94±4.72 (0.00-17.00)	12.30±3.40 (5.00-17.00)	13.12±1.46 (11.00-15.00)
Memory				
**Digit span, forward**	5.00±1.54 (0.00-7.00)	5.00±1.20 (2.00-7.00)	4.09±0.94 (2.00-5.00)	5.62±0.51 (5.00-6.00)
**RAVLT, delayed recall**	2.44±2.66[§] (0.00-8.00)	3.27±3.26[§] (0.00-10.00)	7.10±3.45[♦ #] (0.00-12.00)	3.87±3.27 (0.00-10.00)
**Spatial span, forward**	4.25±1.39 (2.00-7.00)	4.41±1.28 (2.00-7.00)	4.36±1.29 (2.00-6.00)	4.89±0.93 (4.00-7.00)
**Reys complex figure, recall**	7.29±6.19 (0.00-27.50)	9.97±8.03 (0.00-25.00)	9.60±4.91 (4.50-16.50)	8.25±7.32 (0.00-18.50)
Attention and executive function				
**Attentive matrices**	41.38±11.14 (22.00-60.00)	39.42±15.45 (12.00-56.00)	38.18±15.59 (7.00-54.00)	50.37±5.65 (43.00-59.00)
**RCPM**	21.44±7.77 (8.00-35.00)	24.29±9.65 (3.00-36.00)	25.44±8.89 (6.00-34.00)	24.87±4.22 (20.00-32.00)
**Digit span, backward**	3.26±1.23 (0.00-5.00)	3.17±1.46 (0.00-5.00)	2.73±0.79 (2.00-4.00)	4.14±0.69 (3.00-5.00)
**MCST, perseverations**	16.90±12.12[#※] (1.00-44.00)	4.50±4.22[♦] (0.00-13.00)	9.125±9.66 (0.00-25.00)	5.17±7.49[♦] (0.00-20.00)
**TMT, B-A**	199.90±141.13[*※] (67.00-617.85)	115.24±78.70 (26.00-340.00)	108.69±39.94 (55.00-179.53)	101.90±35.86[♦] (43.00-147.00)
Visuospatial abilities				

**Reys complex figure copy**	25.59±9.21 (0.00-36.00)	29.03±6.50 (14.00-35.00)	24.25±12.08 (0.00-34.00)	30.69±3.03 (25.00-35.00)
**Copy of drawings without landmarks**	9.54±2.08 (3.00-12.00)	10.12±1.54 (8.00-12.00)	8.40±2.67 (2.00-11.00)	9.87±0.83 (9.00-11.00)
**Ten-point clock test**	5.28±3.83 (0.00-10.00)	4.83±4.15 (0.00-10.00)	6.54±4.06 (0.00-10.00)	6.25±3.69 (0.00-10.00)
Language & fluency				
**Token test**	26.28±7.95 (5.00-35.00)	26.06±10.06 (4.00-36.00)	27.19±5.93 (14.00-32.00)	29.25±5.28 (19.50-35.00)
**Phonemic fluency**	17.18±11.33 (0.00-47.00)	18.67±10.44 (0.00-31.00)	11.36±7.83 (1.00-25.00)	18.12±7.55 (7.00-33.00)
**Semantic fluency**	19.54±7.25[#] (3.00-30.00)	11.50±7.20[♦ §] (0.00-23.00)	22.09±11.85[#] (0.00-36.00)	21.37±12.21 (0.00-36.00)
Social cognition				
**CATS, affect discrimination**	9.79±1.84 (6.00-12.00)	10.43±1.50 (8.00-12.00)	10.44±1.13 (9.00-12.00)	11.25±1.16 (9.00-12.00)
**CATS, affect naming**	2.56±1.24 (0.00-4.00)	3.29±1.90 (1.00-6.00)	3.22±1.48 (1.00-6.00)	2.50±1.07 (1.00-4.00)
**CATS, affect matching**	6.18±1.68 (3.00-9.00)	7.36±2.13 (4.00-11.00)	6.11±2.85 (0.00-9.00)	5.62±0.92 (4.00-7.00)
**CATS, affect selection**	3.78±1.20 (2.00-6.00)	4.43±1.28 (2.00-6.00)	4.33±1.58 (2.00-6.00)	3.125±0.99 (2.00-4.00)
**SET, intention attribution**	3.52±1.47 (1.00-6.00)	3.83±1.95 (0.00-6.00)	3.80±1.55 (1.00-6.00)	2.00±1.00 (0.00-3.00)
**SET, emotion attribution**	3.33±1.35 (1.00-6.00)	3.42±2.11 (0.00-6.00)	3.90±2.02 (0.00-6.00)	3.29±1.80 (1.00-6.00)
Praxia				
**Goldenbergs test, limb ideomotor apraxia**	32.54±9.39 (0.00-40.00)	35.87±4.13 (28.00-40.00)	32.44±7.67 (14.00-39.00)	37.75±2.96 (33.00-40.00)
**Buccofacial ideomotor apraxia**	17.70±3.34 (9.00-20.00)	17.37±3.96 (8.00-20.00)	16.90±2.33 (13.00-20.00)	18.16±2.79 (13.00-20.00)
Mood & Behaviour				
**FBI, total**	23.00±11.36 (6.00-51.00)	18.07±12.42 (6.00-40.00)	13.00±9.96 (2.00-36.00)	22.12±9.48 (11.00-37.00)

**FBI, A**	14.13±6.50 (4.00-27.00)	11.57±8.20 (2.00-28.00)	9.50±6.30 (2.00-20.00)	13.25±4.46 (8.00-20.00)
**FBI, B**	8.86±7.43 (1.00-24.00)	6.50±5.75 (0.00-20.00)	3.50±4.68 (0.00-16.00)	8.87±6.15 (2.00-17.00)

Values are means ± standard deviations [range]. *p* values refer to ANOVA models, corrected for age, sex and education, followed by post-hoc pairwise comparisons, Bonferroni-corrected for multiple comparisons. The threshold of statistical significance was set at $p<0.05$. ♦ = statistically significant difference with bvFTD; # = statistically significant difference with svPPA; § = statistically significant difference with nfvPPA; ※=statistically significant difference with sbvFTD. Abbreviations: bvFTD = behavioral variant Frontotemporal Dementia; CATS = comprehensive affect testing system; FAB = Frontal assessment battery; FBI = Frontal behavioural inventory; MCST = modified card sorting test; MMSE = Mini-mental-state evaluation; nfvPPA = nonfluent/agrammatic variant Primary Progressive Aphasia; RAVLT=Rey Auditory Verbal Learning Test; RCPM = Ravens Colored Progressive Matrices; sbvFTD = semantic behavioral variant Frontotemporal Dementia; SET = Story-based empathy task; svPPA = semantic variant Primary Progressive Aphasia; TMT = Trail-making test.

Supplementary Table 5. MRI acquisition parameters.

Philips Medical System Ingenia CX 3T scan				
	3D T2-weighted SE	3D FLAIR	3D T1-weighted TFE	Diffusion weighted sequence
**Repetition time (msec)**	2500	4800	7	5900
**Echo time (msec)**	330	267	3.2	78
**Flip angle**	-	90°	9°	-
**Section thickness (mm)**	1	1	1	2.3
**No. of sections**	192	192	204	56
**Matrix**	256x256	256x256	256x240	112x85
**Field of view (mm^2)**	256x256	256x256	256x240	240x232
**Diffusion gradient directions**	-	-	-	6/30/60
***b* value sec/mm^2**	-	-	-	700/1000/2855

Abbreviations: FFE= fast field echo; FLAIR= fluid-attenuated inversion recovery; MRI= magnetic resonance imaging; msec= millisecond; mm= millimeter; No= number; SE=spin echo; sec=second.

Supplementary Table 6. List of brain regions with maximum correlation strength greater than 1.96 standard deviations from FA measures.

**FA**							
**bvFTD**		**svPPA**		**nfvPPA**		**sbvFTD**	
**List of regions**	**R statistic**	**List of regions**	**R statistic**	**List of regions**	**R statistic**	**List of regions**	**R statistic**
**Frontal Sup Orb R**	**0.53**	**Temporal Pole Sup L**	**0.71**	Caudate L	0.42	Amygdala R	0.62
Frontal Sup Medial R	0.46	Temporal Pole Mid L	0.70	**Supplementary Motor Area L**	**0.37**	Temporal Pole Mid R	0.61
Frontal Mid Orb R	0.46	Temporal Inf L	0.66	Pallidum L	0.35	Temporal Inf R	0.59
Frontal Sup R	0.46	Fusiform L	0.56	Frontal Sup L	0.35	Frontal Inf Orb R	0.55
Frontal Mid Orb R	0.44	Amygdala L	0.55	Frontal Sup Medial L	0.34	**Temporal Pole Sup R**	**0.54**
Cingulum Ant R	0.44	Temporal Inf L	0.49	Supplementary Motor Area L	0.34	ParaHippocampal R	0.50
**Frontal Sup Medial L**	**0.43**	Temporal Pole Sup L	0.49			Temporal Pole Mid R	0.49
Cingulum Ant R	0.42	Fusiform L	0.47			Fusiform R	0.48
Frontal Sup Medial R	0.42	Lingual L	0.45			Lingual R	0.47
Rectus R	0.42	Temporal Mid L	0.44			Occipital Inf R	0.45
Frontal Inf Orb R	0.41	Temporal Mid L	0.44			Occipital Inf R	0.45
Frontal Sup Orb L	0.40	Insula L	0.42			Temporal Inf R	0.41
Frontal Sup Medial L	0.40	Calcarine L	0.41			Fusiform R	0.40
Olfactory R	0.39	ParaHippocampal L	0.41			Temporal Inf R	0.40
Cingulum Ant L	0.38						
Frontal Mid Orb L	0.38						
Frontal Inf Orb R	0.38						
Rectus L	0.37						
Frontal Sup R	0.37						
Frontal Sup L	0.36						
Frontal Inf Orb L	0.34						
Frontal Mid R	0.34						
Frontal Mid Orb L	0.34						
Frontal Sup Medial L	0.33						

Abbreviations: Ant= anterior; Inf= inferior; L= left; Mid= middle; Orb= orbitalis; R=right; Sup= superior.

Supplementary Table 7. List of brain regions with maximum correlation strength greater than 1.96 standard deviations from ICVF measures.

ICVF							
**bvFTD**		**svPPA**		**nfvPPA**		**sbvFTD**	
**List of regions**	**R statistic**	**List of regions**	**R statistic**	**List of regions**	**R statistic**	**List of regions**	**R statistic**
**Frontal Sup Orb R**	**0.54**	**Temporal Pole Sup L**	**0.73**	Caudate L	0.41	Amygdala R	0.64
Frontal Sup Medial R	0.47	Temporal Pole Mid L	0.72	**Supplementary Motor Area L**	**0.36**	Temporal Pole Mid R	0.63
Frontal Mid Orb R	0.47	Temporal Inf L	0.70	Frontal Sup L	0.36	Temporal Inf R	0.62
Frontal Sup R	0.46	Fusiform L	0.59	Pallidum L	0.35	**Temporal Pole Sup R**	**0.59**
Cingulum Ant R	0.45	Amygdala L	0.58			Temporal Pole Mid R	0.53
**Frontal Sup Medial L**	**0.44**	Temporal Inf L	0.54			ParaHippocampal R	0.53
Frontal Mid Orb R	0.43	Temporal Pole Sup L	0.52			Frontal Inf Orb R	0.53
Frontal Sup Medial R	0.43	Temporal Mid L	0.50			Fusiform R	0.51
Cingulum Ant R	0.43	Temporal Mid L	0.47			Temporal Inf R	0.46
Frontal Inf Orb R	0.43	Fusiform L	0.46			Temporal Inf R	0.42
Rectus R	0.42	ParaHippocampal L	0.42			Temporal Mid R	0.41
Frontal Sup Medial L	0.39					Occipital Inf R	0.40
Frontal Sup Orb L	0.39					Lingual R	0.40
Olfactory R	0.39						
Frontal Mid Orb L	0.38						
Cingulum Ant L	0.38						
Frontal Sup R	0.37						
Rectus L	0.37						
Frontal Inf Orb R	0.36						
Frontal Sup L	0.35						
Olfactory L	0.34						
Frontal Mid R	0.33						
Frontal Inf Orb L	0.33						
Frontal Sup Medial L	0.32						
Cingulum Mid R	0.32						
Frontal Mid Orb L	0.31						
Frontal Mid R p7	0.31						

Frontal Inf Orb L	0.31						
Cingulum Ant L	0.31						

Abbreviations: Ant= anterior; Inf= inferior; L= left; Mid= middle; Orb= orbitalis; R=right; Sup= superior.

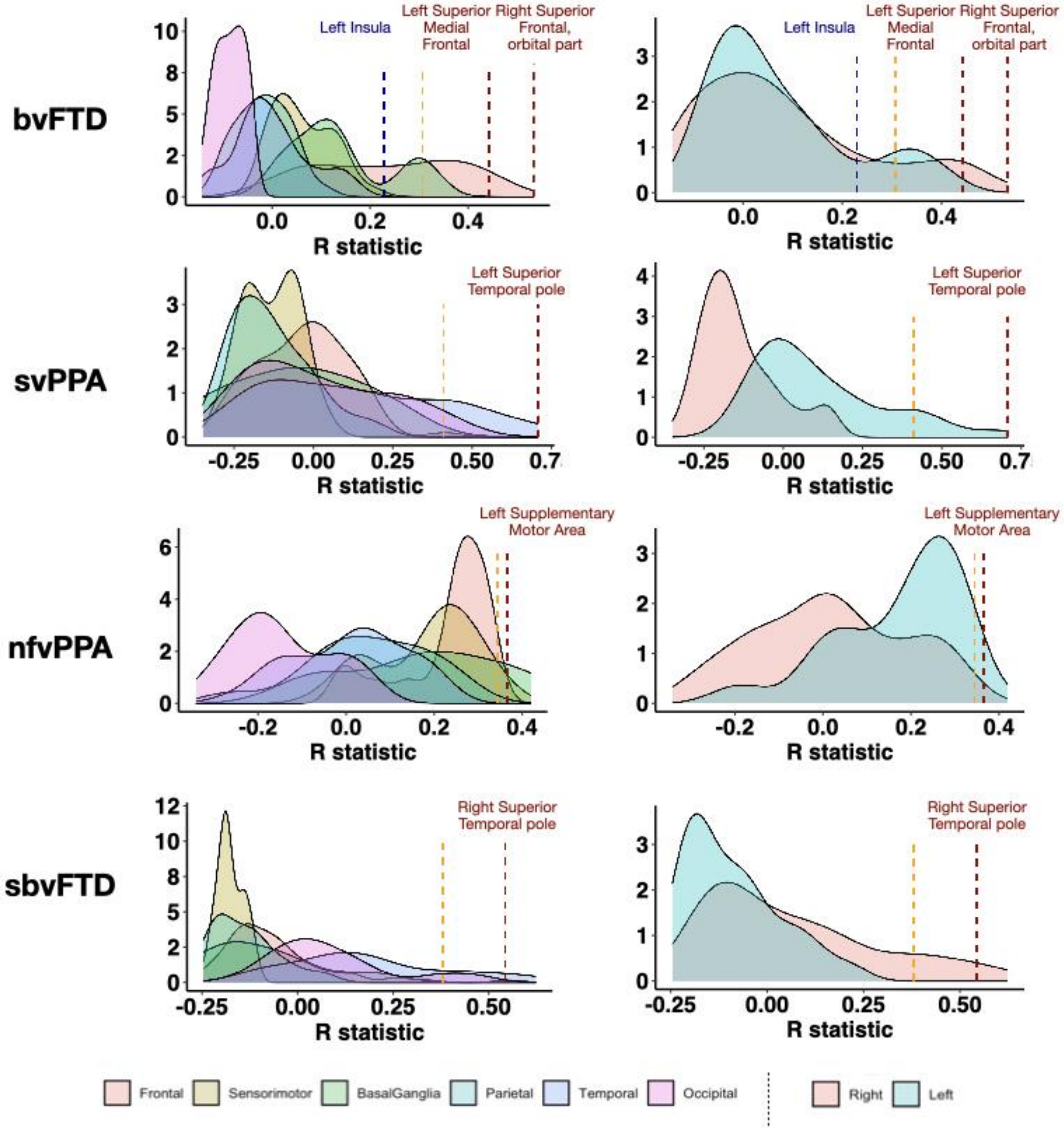

Supplementary Figure 1. Histogram of the maximum R achieved from all ROIs seeded from each phenotype using the FA measure of the structural connectome. The figure is divided into two parts: the left side represents histogram with brain hemispheres, each shown in a different color, and the right side represents different brain lobes, also displayed in distinct colors. For each histogram, a significant cutoff at 1.96 standard deviations (σ) in the upper bound of the tail of the null hypothesis distribution is identified (indicated by dashed orange bars). Different identified disease epicenters, located at the peaks of atrophy in bvFTD (left insula/right superior frontal gyrus (orbital part) and left superior medial frontal cortex), svPPA (left temporal pole), sbvFTD (right temporal pole), and nfvPPA (left supplementary motor area), are marked with dashed dark red or blue lines. Abbreviations: bvFTD = behavioral variant frontotemporal dementia; nfvPPA = nonfluent/agrammatic variant primary progressive aphasia; sbvFTD = semantic behavioral variant frontotemporal dementia; svPPA = semantic variant primary progressive aphasia.
